# Factors influencing cataract awareness and treatment attitudes among the middle-aged and older in western China's rural areas

**DOI:** 10.3389/fpubh.2022.1045336

**Published:** 2023-01-04

**Authors:** Hongyu Guan, Jing Xue, Yuxiu Ding, Yunyun Zhang, Kang Du, Jie Yang

**Affiliations:** ^1^Center for Experimental Economics in Education, Shaanxi Normal University, Xi'an, China; ^2^College of Economics, Xi'an University of Finance and Economics, Xi'an, China

**Keywords:** cataract, associated factors, cataract knowledge, rural areas, western China

## Abstract

**Purpose:**

This study was conducted to determine the level of knowledge about cataracts and the associated factors among adults aged 50 and above in rural areas of Qingcheng county in Gansu Province of Western China, 2020.

**Methods:**

A large community-based cross-sectional study was conducted among the randomly selected 1,503 adults aged 50 and above from October to December 2020. Data were collected by conducting eye examinations and face-to-face interviews. Multivariate binary logistic regression and multivariate linear regression were used to identify associated factors of knowledge about cataracts. Odds Ratio (OR), Coefficient (C), and 95% Confidence Interval (CI) were reported to declare the statistical associations between knowledge about cataracts and the independent variables.

**Results:**

Of the 1,503 study participants, 1,078 (71.7%) had good knowledge about cataracts. The primary school completed [OR = 1.43 (95% CI 1.08–1.90), *P* = 0.012], Secondary school & above [OR = 2.69 (95% CI 1.86–3.89), P <0.001], Examine for cataract [OR = 1.82 (95%CI 1.27–2.62), *P* = 0.001] were positively significantly associated with knowledge about cataracts. Whereas eye examinations [OR = 0.73(95% CI 0.55–0.96), *P* = 0.022] were negatively associated with knowledge about cataracts. Multivariate linear regression analyses showed a significant negative correlation between age and knowledge of cataract treatment options. Living with at least one child was positively correlated with knowledge of the therapeutic effects of cataracts. And monthly household income was significantly positively correlated with knowledge of the therapeutic effects of cataracts, treatment options, and the surgical reimbursement ratio for cataracts.

**Conclusions:**

More than one-third of the participants still had poor knowledge about cataracts. The respondents with at least primary education and above, with previous examinations, and with examination for cataracts had significant associations with good knowledge about cataracts. So, it is recommended that stakeholders in different hierarchies organize health education by considering the education level of the community, focusing on cataract treatment knowledge and the surgical reimbursement ratio to improve cataract surgery rates.

## Introduction

Globally, at least 2.2 billion people have a vision impairment, among which 65.2 million people have moderate or severe distance vision impairment or blindness due to cataracts (1). And the risk of developing cataracts increases with age. In China, from 1990 to 2015, more than one-fifth (about 22%) of people aged 45–89 suffered from any cataract. Around 71% of them were age-related cataract subtypes. By 2050, the number of cataract cases in people aged 45–89 is expected to surpass 240.83 million, representing a prevalence rate of about one-third (2).

Cataracts are subject to an epidemiological transition in the disease burden toward non-communicable diseases and disabilities with an aging population and carry significant individual and societal costs (3). Cataracts present an enormous social and economic burden in many resource-deprived areas, especially remote and impoverished areas in developing countries (4–6). In the case of middle-aged and older adults, vision impairment due to cataracts can contribute to social isolation (7, 8), difficulty in walking (9), a higher risk of falls and fractures (10–12), and a greater likelihood of early admission to nursing or nursing homes (13–15). It may also exacerbate other challenges, such as mobility limitations or cognitive decline (16, 17).

Surgery is the only effective way to treat cataracts and has been rated by the WHO as one of the most cost-effective medical procedures. Cataracts can be safely, efficiently, and cost-efficiently treated with a standard procedure. However, China's cataract surgery rate (CSR) is relatively low compared to developed countries, especially in rural areas. In terms of cataract surgical coverage (CSC), a study in rural China showed that CSC among those with VA worse than 20/200 in both eyes due to cataracts was 62.8% (18), which did not meet the WHO's minimum CSC standard (85%).

Several studies have found that the main barriers to the uptake of cataract surgery are poor knowledge and misunderstandings. People with a better understanding will experience greater certainty about their options and feel more supported, which improves the decision quality for cataract screening and surgery (19). And studies have shown that community-based measures aimed at increasing knowledge of ophthalmic services will significantly lower the burden of cataracts in the region (20).

The previous studies were carried out in provinces and cities with better economic levels or particular terrain (19, 21, 22) and small sample sizes (23, 24). According to the National Bureau of Statistics, 65 and above aged adults reached 176 million in 2019. Consequently, there will be a significant increase in the number of cataract patients in the future, driving the demand for surgery also increase significantly. With the aging of the population and the extension of life expectancy, there will be 700,000 to 1.5 million new cases of farmers with senile cataracts every year. However, evidence of public awareness and knowledge about cataracts is not widely studied in western China's rural areas, especially for people over 50 years old.

Therefore, the present study aims to contribute to the literature by using data from a large-scale cataract study of residents aged 50 and above in rural western China. This study aimed to assess cataract knowledge level and related factors in rural northwest China adults aged 50 and above. Specifically, we pursue three objectives. First, we describe socio-demographic characteristics and knowledge about the cataract of respondents. Second, we estimate factors associated with knowledge about cataracts. Finally, we explore factors related to simple descriptions, treatment options, therapeutic effects, and the surgical reimbursement ratio in knowledge about cataracts.

## Methods

### Setting

The study was conducted following the Declaration of Helsinki and approved by the Stanford University Institutional Review Board (ID. 64279). We obtained the respondents' oral informed consent; all data were analyzed anonymously.

A community-based, cross-sectional study was conducted among adults aged 50 and above in rural areas of Qingcheng county from October to December 2020. The county is located in Gansu Province in Western China and is about 60 km from the city center (Qingyang City). Two public hospitals in Qingcheng have ophthalmology departments with facilities for cataract screening and cataract surgery. Five hospitals in Qingyang city also provide cataract treatment services.

Qingcheng is located in eastern Gansu Province. According to official data, the GDP of Gansu province was 901.67 billion RMB in 2020, ranking 27th among the 31 provincial-level administrative regions in the Chinese mainland. Its per capita GDP was at the bottom among the 31 provinces, and it was the only province whose per capita GDP was lower than 40,000 RMB in China. Compared with other regions in China, Gansu province is a relatively underdeveloped region. Among the 86 districts and counties in Gansu province, Qingcheng ranked 39th with 33,371 RMB per capita GDP in 2020. Our sample area represents the situation in the northwestern rural area.

### Sample

A random sampling procedure at the village level was employed to select the study participants. First, the list of residences in all 153 villages in Qingcheng County was obtained through the County People's Hospital. Ten villages were excluded from the study due to the total population of the village was <800. Then, we randomly selected half of the 143 villages as sample villages and included 73 villages in this study. Finally, all village residents aged 50 and above who attended eye examinations at the village health clinic on the survey day were included as study participants. All adults aged 50 and above who were registered residents of Qingcheng and could give oral informed consent and verbally answer questions on the researcher-administered questionnaire were included in the study. A total of 1,554 participants were eligible for the study, 1,503 of whom provided the complete information requested by the questionnaire to make a response rate of 96.7%.

### Data collection

Data collection included an eye examination and a questionnaire survey.

### Eye examination

Three days before the eye examination, villagers who participated in this study were notified orally by the village health doctor that they could attend the free eye examination at the village clinic between 9 a.m. and 5 p.m. on a specified date. At that time, two questionnaires, an experienced ophthalmologist from Zhongshan Ophthalmic Center, a nurse, and an ophthalmologist from a local public hospital conducted cataract examinations for the villagers who visited the rural clinic. The two stages of an eye exam were vision tests and cataract diagnosis. The nurse first assessed the participant's presenting visual acuity (PVA) using ETDRS (Early Treatment Diabetic Retinopathy Study) charts, a globally recognized test for determining visual acuity. A person would go on to the cataract screens if his or her PVA in the better eye was <0.3 s, the ophthalmologist used a slit lamp microscope and a comprehensive ocular examination with pupil dilatation to identify the cataract. The opinion of the ophthalmologist from the third-class hospital is final when there is a disagreement regarding the diagnosis of cataracts. The nurse and the ophthalmologist, respectively, noted the findings of the visual acuity test and the further cataract diagnosis.

### Questionnaire survey

The questionnaire survey was conducted through a face-to-face interview between the trained enumerators and the participants. Enumerators administered questionnaires to interviewees on basic information, eye care information, and knowledge about cataracts. Basic information includes age, gender, education status, marital status, whether the interviewee lives with at least one child, and household income. Cataract care information was collected through two questions: (1) whether participants had an eye examination in the past, (2) whether participants ever had cataract screens in the past.

The knowledge level of participants was determined based on eight knowledge-related questions that consisted of four domains: simple descriptions, treatment options, therapeutic effects, and the surgical reimbursement ratio. Following the literature (19, 25), these questions were developed and reviewed by a group of health experts from Shaanxi Normal University and Zhongshan Ophthalmic Center, an authoritative ophthalmology institution in China. One point for each correct answer. Participants' overall knowledge was categorized using the mean score as good if the score was between 4 and 8 points and poor if the score was <4. In the eight knowledge-related questions, we added the scores of the two questions with a simple description of cataracts to get the participant's knowledge level in simple descriptions of cataracts. Knowledge of treatment options and therapeutic effects was also measured in this way. We used the total scores of these there domains in our following statistical analyses. The last domain of knowledge about cataracts, surgical reimbursement ratio, measures whether adults aged 50 and above were informed of the reimbursement ratio for cataract surgery.

### Statistical analysis

All analyses were conducted using Stata16 (StataCorp). Descriptive statistics such as percent and frequency were calculated. Multivariate binary logistic regression analysis was employed to determine factors linked with the participants' knowledge of cataracts and the surgery reimbursement ratio, which are binary outcome variables. Multivariate linear regression analysis was employed to identify characteristics linked with awareness of cataract treatment options, simple descriptions, and therapeutic effects, which are continuous outcome variables. Based on previous literature (26, 27), variables, including age, gender, education status, marital status, whether the participant lives with at least one child, household income, and whether the participant has a cataract, previous eye examination, and examination for cataract, were included in the regression analyses. *P* < 0.05 was considered statistically significant in this study. Odds Ratio (OR), Coefficient (C), and 95% Confidence Interval (CI) were reported to declare the statistical associations between knowledge about cataracts and the independent variables.

## Results

### Socio-demographic characteristics of participants

Table 1 presents the descriptive statistics for the sample participants. Among the 1,554 participants, 1,503 of them have completed the interview, yielding a response rate of 96.7%. The median (±IQR) age was 62 (±13 years). Among the sample participants, 891 (59.2%) were females, 883 (58.7%) didn't live with any of their offspring, and 1,156 (76.9%) had completed primary education. About three-quarters of the study participants have a spouse, and more than half do not live with a child. Seventy-one percent (1,058/1,503) of the study participants' monthly household income was <1,000 RMB (Criteria for low-income families in rural China, 137.60 USD). In terms of the cataract, 824 (54.8%) of our participants had cataract symptoms, 966 (64.3%) of them had a previous eye examination, and 1,232 (82.0%) had a history of cataract screening (Table 1).

**Table 1:** Socio-demographic characteristics of adults aged 50 and above in rural western China (*n* = 1,503).

**Variables**	**Frequency**	**Percent (%)**
**Age**		
50–59	645	42.9
60–69	521	34.7
70 and above	337	22.4
**Gender**		
Male	612	40.72
Female	891	59.28
**Education status**		
No formal education	658	43.78
Primary education	498	33.13
Secondary education and above	347	23.09
Marital status		
Don't have a spouse	373	24.80
Have a spouse	1,130	75.20
**Live with a least one child**		
Yes	620	41.3
No	883	58.7
**Monthly household income (RMB)**		
≤ 1,000	1,058	70.4
1,000–2,000	276	18.4
>2,000	169	11.2
**Cataract**		
Yes	679	45.2
No	824	54.8
**Previous eye examination**		
Yes	537	35.7
No	966	64.3
**Examine for cataracts**		
Yes	271	18.0
No	1,232	82.0

### Participants' knowledge regarding cataract

In our study, more than two-thirds (1,039, 69.1%) of the participants believed cataract was a common disease in older adults, and 1,232 (82.0%) of the participants recognized the importance of regular cataract checkups.

Regarding treatment options, 717 (47.7%) participants mistakenly thought that cataracts could be cured by taking medicine or using eye drops. More than 70% of participants understood that surgery was safe, and more than 1,093 (72.7%) said it was a successful treatment. 67.9% said having cataract surgery can enhance vision. However, nearly 64% (959) of participants perceived that cataracts have to be treated after they can't see clearly. Moreover, the vast majority, 1,465 (97.5%) participants, did not know the reimbursement policy for cataract surgery. In general, the mean (±SD) knowledge score point was 4.49 ± 1.90 points.

After the knowledge about cataracts was subdivided, there were two questions to determine the participants' knowledge of simple descriptions of cataracts. The average score of participants' knowledge of simple descriptions of cataracts was 1.511, the highest score was 2, and the lowest score was 0. Similarly, participants' knowledge of treatment options for cataracts averaged 1.566, with the highest and lowest scores being 3 and 0, respectively. Participants' knowledge of the therapeutic effects of cataracts averaged 1.566, with a maximum score of 2. In terms of knowledge of the surgical reimbursement ratio of cataracts, the vast majority, 1,465 (97.5%) participants did not know the reimbursement rate for cataract surgery. Of the 1,503 participants, more than half (61.9%) had a good overall knowledge about cataracts, meaning an overall knowledge score of more than 4 points (Table 2).

**Table 2:** Participants' knowledge about cataracts of adults aged 50 and above in rural western China 2021.

**Variables**	**Responses**	
**Panel A: eight questions related to cataract knowledge**		
	Yes n (%)	No n (%)
Is cataract a common disease in older adults?	1,039 (69.1)	464 (30.9)
Do people over 50 need regular cataract checkups?	1,232 (82.0)	271 (18.0)
Can medicine or eye drops cure cataracts?	717 (47.7)	786 (52.3)
Can surgery cure cataracts?	1,093 (72.7)	410 (27.3)
Do cataracts have to be treated after they can't see clearly?	544 (36.2)	959 (63.8)
Is cataract surgery safe?	1,066 (70.9)	437 (29.1)
Can receiving cataract surgery improve vision?	1,021 (67.9)	482 (32.1)
Do you know what the reimbursement ratio for cataract surgery is?	38 (2.5)	1,465 (97.5)
**Panel B: participants' overall knowledge about cataracts and knowledge of four domains**		
	Mean/N	Min, Max/%
Simple descriptions	1.511	0,2
Treatment options	1.566	0,3
Therapeutic effects	1.389	0,2
Surgical reimbursement ratio		
Yes	38	2.5
No	1465	97.5
Knowledge about cataract		
Good	931	61.9
Poor	572	38.1

### Factors associated with knowledge about cataracts

The result of multivariate binary logistic regression analysis showed that higher education status (*P* < 0.05) and having been examined for cataracts (*P* < 0.01) were significantly associated with better knowledge about cataracts. Additionally, participants with previous eye examinations (*P* < 0.05) had significantly worse knowledge about cataracts (Table 3).

**Table 3:** Factors associated with knowledge about cataracts of adults aged 50 and above in rural western China 2021.

**Variables**	**Knowledge about cataracts**				**OR (95% CI)**	***P*-value**
	**Poor**		**Good**			
	**No**.	**(%)**	**No**.	**(%)**		
**Age**						
50–59	149	(23.1)	496	(76.9)	1	-
60–69	152	(29.2)	369	(70.8)	0.93 (0.69–1.27)	0.659
70 and above	124	(36.8)	213	(63.2)	0.70 (0.49–1.01)	0.057
**Gender**						
Male	152	(24.8)	460	(75.2)	1	-
Female	273	(30.6)	618	(69.4)	0.92 (0.71–1.20)	0.545
**Education status**						
No formal education	238	(36.2)	420	(63.8)	1	-
Primary education	133	(26.7)	365	(73.3)	1.43 (1.08–1.90)	0.012
Secondary education and above	54	(15.6)	293	(84.4)	2.69 (1.86–3.89)	< 0.001
**Marital status**						
Don't have a spouse	115	(30.8)	258	(69.2)	1	-
Have a spouse	310	(27.4)	820	(72.6)	1.04 (0.79–1.37)	0.768
**Live with a least one child**						
Yes	158	(25.5)	462	(74.5)	1.27 (0.99–1.62)	0.058
No	267	(30.2)	616	(69.8)	1	-
**Monthly household income (RMB)**						
≤ 1,000	322	(30.4)	736	(69.6)	1	-
1,000–2,000	71	(25.7)	205	(74.3)	1.10 (0.80–1.50)	0.571
>2,000	32	(18.9)	137	(81.1)	1.42 (0.94–2.16)	0.096
**Cataract**						
Yes	227	(33.4)	452	(66.6)	0.80 (0.61–1.05)	0.102
No	198	(24.0)	626	(76.0)	1	-
**Previous eye examination**						
Yes	158	(29.4)	379	(70.6)	0.73 (0.55–0.96)	0.022
No	267	(27.6)	699	(72.4)	1	-
**Examine for cataracts**						
Yes	66	(24.4)	205	(75.6)	1.82 (1.27–2.62)	0.001
No	359	(29.1)	873	(70.9)	1	-

### Factors associated with simple descriptions, treatment options for cataracts

Participants with higher education status (*P* < 0.05), having examined for cataracts (*P* < 0.05), had significantly better knowledge of simple descriptions of cataracts. However, having previous eye examinations (*P* < 0.05) was associated with worse knowledge of simple descriptions of cataracts. In the multivariate linear regression model, the following characteristics were associated with knowledge of treatment options for cataracts: older age (*P* < 0.001); having primary education (*P* < 0.01); having secondary education and above (*P* < 0.001); having monthly household income between 1,000 and 2,000 (*P* < 0.01); having monthly household income >2,000 (*P* < 0.01); having examined for cataract (*P* < 0.001). Among them, participants of older age were associated with worse knowledge of treatment options for cataracts. They were more likely to choose the wrong treatment options for cataracts (Table 4).

**Table 4:** Factors associated with simple descriptions, treatment options for cataracts of adults aged 50 and above in rural western China 2021.

**Variables**	**Simple descriptions** ^**a**^		**Treatment options** ^**b**^	
	**C^c^ (95% CI)**	***P*-value**	**C^c^ (95% CI)**	***P*-value**
**Age**				
50–59	1	-	1	-
60–69	−0.00 (−0.09–0.08)	0.987	−0.22 (−0.35−0.10)	< 0.001
70 and above	−0.10 (−0.021–0.01)	0.067	−0.31 (−0.47−0.16)	< 0.001
**Gender**				
Male	1	–	1	-
Female	−0.04 (−0.12–0.03)	0.269	−0.02 (−0.13–0.09)	0.748
**Education status**				
No formal education	1	–	1	-
Primary education	0.11 (0.02–0.19)	0.014	0.16 (0.04–0.28)	0.008
Secondary education and above	0.28 (0.18–0.38)	< 0.001	0.34 (0.20–0.48)	< 0.001
**Marital status**				
Don't have a spouse	1	–	1	-
Have a spouse	0.01 (−0.07–0.09)	0.839	0.00 (−0.11–0.12)	0.963
Live with a least one child				
Yes	0.03 (−0.04–0.10)	0.438	0.05 (−0.05–0.15)	0.360
No	1	–	1	-
**Monthly household income (RMB)**				
≤ 1,000	1	–	1	-
1,000–2,000	−0.02 (−0.11–0.07)	0.651	0.17 (0.04–0.30)	0.009
>2,000	0.05 (−0.06–0.16)	0.412	0.21 (0.06–0.38)	0.007
**Cataract**				
Yes	−0.03 (−0.11–0.05)	0.429	−0.03 (−0.15–0.08)	0.582
No	1	–	1	-
**Previous eye examination**				
Yes	−0.09 (−0.17−0.01)	0.026	−0.07 (−0.18–0.04)	0.260
No	1	–	1	-
**Examine for cataracts**				
Yes	0.12 (0.01–0.22)	0.027	0.28 (0.14–0.43)	< 0.001
No	1	–	1	-

a Questionnaire asked respondents to judge questions that simple descriptions of cataracts, including the following two questions: 1. Is cataract a common disease in older adults? 2. Do people over 50 need regular cataract checkups? One point for each correct answer.

b Questionnaire asked respondents to judge questions that treatment options for cataracts, including the following three questions: 1. Can taking medicine or eye drops cure cataracts? 2. Can surgery cure cataracts? 3. Do cataracts have to be treated after they can't see clearly? One point for each correct answer.

c The coefficient (C) in the multivariate linear regression analysis.

### Factors associated with therapeutic effects, the surgical reimbursement ratio for cataracts

In multivariate linear regression models (Table 5), we found that having secondary education and above (*P* < 0.01), living with at least one child (*P* < 0.01), monthly household income (RMB) >2,000 (*P* < 0.01), having examined for cataract (*P* < 0.01) were associated with better knowledge of therapeutic effects of cataract, while previous eye examination was associated with worse knowledge of therapeutic effects of cataract (*P* < 0.01). Monthly household income (RMB) >2,000 (*P* < 0.05) and having been examined for cataracts (*P* < 0.05) were associated with a better understanding of the surgical reimbursement ratio for cataracts.

**Table 5:** Factors associated with therapeutic effects, surgical reimbursement ratio for cataracts of adults aged 50 and above in rural western China 2021.

**Variables**	**Therapeutic effects** ^**a**^		**Surgical reimbursement ratio** ^**b**^	
	**C^c^ (95% CI)**	***P*-value**	**OR (95% CI)**	***P*-value**
**Age**				
50–59	1	-	1	-
60–69	0.05 (−0.05–0.15)	0.302	0.83 (0.40–1.71)	0.613
70 and above	−0.07 (−0.20–0.05)	0.228	0.77 (0.28–2.09)	0.605
**Gender**				
Male	1	–	1	–
Female	−0.06 (−0.14–0.03)	0.204	0.67 (0.34–1.35)	0.266
**Education status**				
No formal education	1	–	1	–
Primary education	0.08 (−0.02–0.18)	0.107	1.58 (0.68–3.68)	0.292
Secondary education and above	0.20 (0.08–0.31)	0.001	1.55 (0.62–3.87)	0.352
**Marital status**				
Don't have a spouse	1	–	1	–
Have a spouse	0.09 (0.01–0.18)	0.076	0.53 (0.27–1.08)	0.079
**Live with a least one child**				
Yes	0.11 (0.03–0.19)	0.009	0.86 (0.44–1.68)	0.656
No	1	–	1	–
**Monthly household income (RMB)**				
≤ 1,000	1	–	1	–
1,000–2,000	0.03 (−0.08–0.13)	0.636	1.74 (0.74–4.08)	0.204
>2,000	0.20 (0.06–0.33)	0.003	2.89 (1.23–6.76)	0.015
Cataract				
Yes	−0.05 (−0.15–0.04)	0.269	0.51 (0.26–1.00)	0.050
No	1	–	1	–
**Previous eye examination**				
Yes	−0.10 (−0.19–0.00)	0.048	2.09 (0.90–4.82)	0.084
No	1	–	1	–
**Examine for cataracts**				
Yes	0.20 (0.08–0.32)	0.001	2.41 (1.05–5.55)	0.038
No	1	–	1	–

a Questionnaire asked respondents to judge questions that the therapeutic effect of cataracts, including the following two questions: 1. Is cataract surgery safe? 2. Can cataract surgery improve vision? One point for each correct answer.

b Questionnaire asked respondents to answer a question: do you know what the reimbursement ratio for cataract surgery is?

c The coefficient (C) in the multivariate linear regression analysis.

## Discussion

This current study provided vital epidemiological data for assessing cataract knowledge level and related factors in adults aged 50 and above in rural northwest China. This study result revealed that nearly 40% of our participants did not have sufficient knowledge about cataracts, especially regarding treatment options and surgical reimbursement ratio.

The finding that participants with higher levels of education had better knowledge about cataracts can be attributed to their better knowledge, and, therefore, more appropriate health-seeking behavior (28). Regarding cataract care information, those participants who had been examined for cataracts were 1.8 times more knowledgeable than those without an examination. This finding is supported by studies done in Ethiopia (27, 29). This might be explained by the possibility that a person who has already undergone a cataract examination may learn more from medical professionals, other patients, or attendants. Participants in the study who had previously experienced eye exams were 27% less likely to have better knowledge than those who had not. This could result from the adverse effects of inadequate information on health status, which causes visits for eye exams to be more frequent. However, because of the nature of cross-sectional data, we were unable to identify this causal relationship, which was one of the limitations of our study. Moreover, a simple eye examination is limited in improving knowledge (30, 31).

After dividing cataract knowledge into four aspects, we noticed that participants with a higher monthly household income had better knowledge of treatment options, therapeutic effects, and surgical reimbursement ratio for cataracts. This may be because that people with better economic levels would have more opportunities to access eye care services. In contrast, lower-income people may fear going to an eye hospital as they assume it would be exorbitant (32). Meanwhile, our study found that participants aged 60 and above were less likely to have good knowledge of treatment options for cataracts compared with a younger age group. Possibly due to fragile physical conditions and increasing age, people aged 60 and above generally lack access to group health talks and community health education sessions routinely organized in the community (33). The study result also showed that living with at least one child was associated with better knowledge of the therapeutic effects of cataracts. This may be due to family as the most common effective source of knowledge (34, 35).

This study also has some limitations. First, self-reported recall data relies on the reliability of informants. The utilization of eye care services is a self-reported variable, and self-report bias may influence our results (36, 37); this issue may be greater when older people are involved, as in the present case. Second, this study had a cross-sectional design; therefore, we failed to establish a causal relationship between factors. Despite these limitations, this study has its strength of the large sample size with a high response rate. And we believe that our research provides new and helpful information to inform policy.

## Conclusions

More than one-third of the participants still had poor knowledge about cataracts. The respondents with at least primary education and above, with previous examinations, and with cataract examination had significant associations with good knowledge about cataracts. However, a considerable knowledge gap regarding the treatment options and surgical reimbursement ratio was recognized. Hence, it might be logical to pay special attention to implementing health education in rural communities, especially regarding cataract treatment. Policymakers should include eye screening in the fundamental public service system and give attention to the availability and accessibility of the primary eye care unit. Second, policymakers should organize different health education by considering the education level of the community, focusing on cataract treatment knowledge and surgical reimbursement ratio to improve cataract surgery rates. Third, national and regional ministries of health offices should organize different health education programs focusing on risk factors and various prevention methods to delay the occurrence of the disease.

## Data availability statement

The original contributions presented in the study are included in the article/supplementary material, further inquiries can be directed to the corresponding author.

## Ethics statement

The studies involving human participants were reviewed and approved by Declaration of Helsinki Stanford University Institutional Review Board (Palo Alto, USA; ID 64729). The patients/participants provided their written informed consent to participate in this study.

## Author contributions

HG and KD designed the study. JX, YZ, YD, and KD collected the data. JX performed the statistical analyses. JX, JY, and HG drafted the manuscript. All authors interpreted the results, made critical revisions, provided intellectual content to the manuscript, approved the final version, and agreed to be accountable for all aspects of this work.

## References

[1] World Health Organization. World Report on Vision. Geneva: World Health Organization. (2019). p. 160. Available online at: https://apps.who.int/iris/handle/10665/328717 (accessed December 13, 2022).

[2] Song P Wang H Theodoratou E Chan KY Rudan I. The national and subnational prevalence of cataract and cataract blindness in China: a systematic review and meta-analysis. J Glob Health. (2018) 8:010804. 10.7189/jogh.08.010804 29977532 PMC6005639

[3] Pawar S,. Causes of Blindness Vision Impairment in 2020 Trends over 30 Years, Prevalence of Avoidable Blindness in Relation to VISION 2020: The Right to Sight: An Analysis for the Global Burden of Disease Study. (2021). Available online at: https://www.ssrn.com/abstract=3939242 10.2139/ssrn.3939242 (accessed December 13, 2022). PMC7820391 33275949

[4] Nirmalan PK. Utilisation of eye care services in rural south India: the Aravind Comprehensive Eye Survey. Br J Ophthalmol. (2004) 88:1237–41. 10.1136/bjo.2004.042606 15377541 PMC1772350

[5] Rabiu MM. Cataract blindness and barriers to uptake of cataract surgery in a rural community of northern Nigeria. Br J Ophthalmol. (2001) 85:776–80. 10.1136/bjo.85.7.776 11423446 PMC1724057

[6] Yin Q Hu A Liang Y Zhang J He M Lam DSC. A Two-site, population-based study of barriers to cataract surgery in rural China. Invest Ophthalmol Vis Sci. (2009) 50:1069. 10.1167/iovs.08-2783 19029020

[7] Evans RL. Loneliness, depression, and social activity after determination of legal blindness. Psychol Rep. (1983) 52:603–8. 10.2466/pr0.1983.52.2.603 6878556

[8] Verstraten PFJ Brinkmann WLJH Stevens NL Schouten JSAG. Loneliness, adaptation to vision impairment, social support and depression among visually impaired elderly. Int Congress Series. (2005) 1282:317–21. 10.1016/j.ics.2005.04.017

[9] Swenor BK Munoz B West SK. A longitudinal study of the association between visual impairment and mobility performance in older adults: the Salisbury eye evaluation study. Am J Epidemiol. (2014) 179:313–22. 10.1093/aje/kwt257 24148711 PMC3954103

[10] Lord SR Dayhew J. Visual risk factors for falls in older people. J Am Geriatr Soc. (2001) 49:508–15. 10.1046/j.1532-5415.2001.49107.x 11380741

[11] Menezes C Vilaça KHC Menezes RL. Falls and quality of life of people with cataracts. Revista Brasileira de Oftalmologia. (2016) 75:1. 10.5935/0034-7280.20160009

[12] Hong T Mitchell P Burlutsky G Samarawickrama C Wang JJ. Visual impairment and the incidence of falls and fractures among older people: longitudinal findings from the blue mountains eye study. Invest Ophthalmol Vis Sci. (2014) 55:7589. 10.1167/iovs.14-14262 25370514

[13] Friedman DS. Racial variations in causes of vision loss in nursing homes: the salisbury eye evaluation in nursing home groups (SEEING) study. Arch Ophthalmol. (2004) 122:1019. 10.1001/archopht.122.7.1019 15249367

[14] Mitchell P Hayes P Wang JJ. Visual impairment in nursing home residents: the blue mountains eye study. Medical J Australia. (1997) 166:73–6. 10.5694/j.1326-5377.1997.tb138724.x 9033561

[15] Owsley C. The visual status of older persons residing in nursing homes. Arch Ophthalmol. (2007) 125:925. 10.1001/archopht.125.7.925 17620572

[16] Guthrie DM Davidson JGS Williams N Campos J Hunter K Mick P. Combined impairments in vision, hearing and cognition are associated with greater levels of functional and communication difficulties than cognitive impairment alone: Analysis of interRAI data for home care and long-term care recipients in Ontario. PLoS ONE. (2018) 13:e0192971. 10.1371/journal.pone.0192971 29447253 PMC5814012

[17] Bowen M Edgar DF Hancock B Haque S Shah R Buchanan S. The prevalence of visual impairment in people with dementia (the PrOVIDe study): a cross-sectional study of people aged 60–89 years with dementia and qualitative exploration of individual, carer and professional perspectives. Health Serv Deliv Res. (2016) 4:1–200. 10.3310/hsdr04210 27489923

[18] Zhao J Xu X Ellwein LB Guan H He M Liu P. Cataract surgical coverage and visual acuity outcomes in rural China in 2014 and comparisons with the 2006 China nine-province survey. Am J Ophthalmol. (2018) 193:62–70. 10.1016/j.ajo.2018.06.004 29906431 PMC6109593

[19] Ye G Qu B Shi W Chen X Ma P Zhong Y. Knowledge about benefits and risks of undergoing cataract surgery among cataract patients in Southern China. Int Ophthalmol. (2020) 40:2889–99. 10.1007/s10792-020-01473-7 32601961

[20] Athanasiov PA Casson RJ Newland HS Shein WK Muecke JS Selva D. Cataract surgical coverage and self-reported barriers to cataract surgery in a rural Myanmar population. Clin Experiment Ophthalmol. (2008) 36:521–5. 10.1111/j.1442-9071.2008.01829.x 18954313

[21] Li LS. Knowledge, attitudes and practices related to seeking medical eyecare services by adults with moderate-to-severe visual impairment in rural Yueqing, Wenzhou, China: a cross-sectional survey. Int J Ophthalmol. (2020) 13:1115–23. 10.18240/ijo.2020.07.16 32685401 PMC7321945

[22] Bassett KL. Cataract surgical coverage and outcome in the Tibet Autonomous Region of China. Br J Ophthalmol. (2005) 89:5–9. 10.1136/bjo.2004.048744 15615736 PMC1772483

[23] Lau JTF. Knowledge about cataract, glaucoma, and age related macular degeneration in the Hong Kong Chinese population. Br J Ophthalmol. (2002) 86:1080–4. 10.1136/bjo.86.10.1080 12234882 PMC1771305

[24] Xu Y He J Lin S Zhang B Zhu J Resnikoff S. General analysis of factors influencing cataract surgery practice in Shanghai residents. BMC Ophthalmol. (2018) 18:102. 10.1186/s12886-018-0767-5 29669533 PMC5907364

[25] Chen T Jin L Zhu W Wang C Zhang G Wang X. Knowledge, attitudes and eye health-seeking behaviours in a population-based sample of people with diabetes in rural China. Br J Ophthalmol. (2021) 105:806–11. 10.1136/bjophthalmol-2020-316105 32737033

[26] Du K Guan H Zhang Y Ding Y Wang D. Knowledge of cataracts and eye care utilization among adults aged 50 and above in rural Western China. Front Public Health. (2022) 10:1034314. 10.3389/fpubh.2022.1034314 36457322 PMC9706408

[27] Fikrie A Mariam YG Amaje E Bekele H. Knowledge about cataract and associated factors among adults in Yirgalem town, Sidama National Regional State, southern Ethiopia, 2020: a community based cross sectional study design. BMC Ophthalmol. (2021) 21:79. 10.1186/s12886-021-01844-3 33568076 PMC7877085

[28] Fotouhi A Hashemi H Mohammad K. Eye care utilization patterns in Tehran population: a population based cross-sectional study. BMC Ophthalmol. (2006) 6:4. 10.1186/1471-2415-6-4 16423308 PMC1382253

[29] Alimaw YA Hussen MS Tefera TK Yibekal BT. Knowledge about cataract and associated factors among adults in Gondar town, northwest Ethiopia. PLoS ONE. (2019) 14:e0215809. 10.1371/journal.pone.0215809 31013319 PMC6478322

[30] Kemper AR Helfrich A Talbot J Patel N Crews JE. Improving the rate of preschool vision screening: an interrupted time-series analysis. Pediatrics. (2011) 128:e1279–84. 10.1542/peds.2010-3679 21987706

[31] Yawn BP Kurland M Butterfield L Johnson B. Barriers to seeking care following school vision screening in Rochester, Minnesota. J School Health. (1998) 68:319–24. 10.1111/j.1746-1561.1998.tb00592.x 9800181

[32] Vela C Samson E Zunzunegui MV Haddad S Aubin MJ Freeman EE. Eye care utilization by older adults in low, middle, and high income countries. BMC Ophthalmol. (2012) 12:5. 10.1186/1471-2415-12-5 22471351 PMC3378437

[33] Ko KK Pumpaibool T Wynn MMM Win Y Kyi TM Aung PL. Door-to-door eye health education to improve knowledge, attitude, and uptake of eyecare services among elderly with cataracts: a quasi-experimental study in the central tropical Region, Myanmar. OPTH. (2021) 15:815–24. 10.2147/OPTH.S287257 33658757 PMC7920616

[34] Pardhan S Mughal N Mahomed I. Self-reported eye disease in elderly South Asian subjects from an inner city cluster in Bradford: a small-scale study to investigate knowledge and awareness of ocular disease. Eye. (2000) 14:620–4. 10.1038/eye.2000.153 11040910

[35] Haddad MF Bakkar MM Abdo N. Public awareness of common eye diseases in Jordan. BMC Ophthalmol. (2017) 17:177. 10.1186/s12886-017-0575-3 28969614 PMC5625650

[36] Kobayashi T Boase J. No Such Effect? The implications of measurement error in self-report measures of mobile communication use communication methods and measures. Commun Methods Meas. (2012) 6:126–43. 10.1080/19312458.2012.679243

[37] Scharkow M. The accuracy of self-reported internet use—a validation study using client log data. Commun Methods Meas. (2016) 10:13–27. 10.1080/19312458.2015.1118446

